# Growth Hormone-Releasing Peptide 6 Enhances the Healing Process and Improves the Esthetic Outcome of the Wounds

**DOI:** 10.1155/2016/4361702

**Published:** 2016-04-20

**Authors:** Yssel Mendoza Marí, Maday Fernández Mayola, Ana Aguilera Barreto, Ariana García Ojalvo, Yilian Bermúdez Alvarez, Ana Janet Mir Benítez, Jorge Berlanga Acosta

**Affiliations:** ^1^Wound Healing and Cytoprotection Group, Biomedical Research Direction, Center for Genetic Engineering and Biotechnology, Avenue 31/158 and 190, P.O. Box 6162, Playa, 10600 Havana, Cuba; ^2^Formulation Department, Technological Development Direction, Center for Genetic Engineering and Biotechnology, Avenue 31/158 and 190, P.O. Box 6162, Playa, 10600 Havana, Cuba; ^3^Esthetic Surgery Department, “Joaquín Albarrán” Hospital, Avenue 26 and Boyeros, Plaza de la Revolución, 10600 Havana, Cuba

## Abstract

In addition to its cytoprotective effects, growth hormone-releasing peptide 6 (GHRP-6) proved to reduce liver fibrotic induration. CD36 as one of the GHRP-6 receptors appears abundantly represented in cutaneous wounds granulation tissue. The healing response in a scenario of CD36 agonistic stimulation had not been previously investigated. Excisional full-thickness wounds (6 mmØ) were created in the dorsum of Wistar rats and topically treated twice a day for 5 days. The universal model of rabbit's ears hypertrophic scars was implemented and the animals were treated daily for 30 days. Treatments for both species were based on a CMC jelly composition containing GHRP-6 400 *μ*g/mL. Wounds response characterization included closure dynamic, RT-PCR transcriptional profile, histology, and histomorphometric procedures. The rats experiment indicated that GHRP-6 pharmacodynamics involves attenuation of immunoinflammatory mediators, their effector cells, and the reduction of the expression of fibrotic cytokines. Importantly, in the hypertrophic scars rabbit's model, GHRP-6 intervention dramatically reduced the onset of exuberant scars by activating PPAR*γ* and reducing the expression of fibrogenic cytokines. GHRP-6 showed no effect on the reversion of consolidated lesions. This evidence supports the notion that CD36 is an active and pharmacologically approachable receptor to attenuate wound inflammation and accelerate its closure so as to improve wound esthetic.

## 1. Introduction

Hypertrophic scarring is a form of abnormal, exuberant healing, locally aggressive, and recurrent cutaneous fibroproliferative condition, characterized by excessive extracellular matrix (ECM) accumulation during the cutaneous healing process. Including keloids and hypertrophic scars (HTS), these aberrant processes lead to esthetically disfiguring scars, patients' psychological stress, and functional impairment [1]. The cellular and molecular mechanisms underlying the formation of these raised dermal scars are poorly understood. Recent whole genome profiling and proteomic studies have led to the identification of regulatory elements with different expression profiles in HTS and keloid tissues [2]. The limited understanding of the pathophysiology of these processes has led to investigating a broad spectrum of potential antihypertrophic scarring candidates [3].

Triamcinolone acetonide (TA) has long been the steroid of choice for the treatment of skin fibrotic disorders, providing the best relief of local symptoms such as scars flattening. Nevertheless, TA is associated with adverse events such as dermal atrophy, telangiectasia, and immunosuppression [4, 5]. Despite the multitude of therapeutic strategies to prevent or reduce keloid and HTS formation, these conditions remain as orphan clinical niches of ultimately effective interventions [6].

Our group recently demonstrated the antifibrotic effects of the growth hormone-releasing peptide 6 (GHRP-6) in a rat model of liver cirrhosis. GHRP-6 prevented parenchymal fibrotic induration in more than 85% and removed in about 75% the accumulated fibrotic material in both preventive and therapeutic administration schemes. Differentially expressed genes in a microarray experiment indicated that GHRP-6 modulates the expression of genes involved in the redox metabolism, as in the mesenchymal cells response to injury [7].

During the last 15 years, a plethora of experimental evidence supports the pharmacological benefits of the exogenous administration of synthetic growth hormone-releasing peptides (GHRPs). In parallel to their growth hormone-releasing action, these agents exert cytoprotective effects encompassing cardiac and extracardiac organs [8]. GHRP-6 is a class of peptidyl GH secretagogue, similar to met-enkephalin, that has reproducibly shown antinecrogenic and antiapoptotic properties in multiple experimental scenarios, including ischemia/reperfusion [9–11]. Globally speaking, exogenously administered GHRP-6 has broadly been shown to act as a prosurvival factor for cells and tissues threatened by otherwise lethal insults.

More than a decade ago, CD36 was identified as one of the GHRP-6 receptors [12]. This is a scavenger receptor endowed with multiligand and multifunctional capabilities and is expressed by a broad constellation of mammalian cells [13]. Granulation tissue neovascularization is perhaps the most renowned physiological role of CD36 in wound healing [14]. Serendipitous observations of our laboratory indicated that CD36 mRNA transcript appeared abundantly represented in clinical samples of granulation tissue of either acute (deep burn injuries) or chronic (pressure ulcers) wounds, as in laboratory rat's controlled full-thickness wounds. This finding incited us to speculate on the effects associated with CD36 agonistic stimulation beyond that of the angiostatic action via thrombospondin binding [15]. Here we provide the first experimental evidence on the favorable impact of the topical administration of GHRP-6, as a candidate to qualitatively improve the healing process.

## 2. Materials and Methods

### 2.1. Ethics

The experiments were conducted following the approval by the institutional Animal Welfare Committee. All the procedures were conducted following the internal standards of animal care and protection established by the Animal Facility Core of the Center for Genetic Engineering and Biotechnology, Havana, Cuba.

### 2.2. GHRP-6 Formulation and Treatments

The hexapeptide GHRP-6 (His-d-Trp-Ala-Trp-d-Phe-Lys-NH2) was purchased from BCN Peptides (Barcelona, Spain). Fresh preparations were obtained by diluting the peptide in sterile 1% sodium carboxymethylcellulose- (CMC-) based jelly formulation to a final concentration of 400 *μ*g/mL.

### 2.3. Wound Healing in Rats

Healthy male Wistar rats (250–270 g) were purchased from the National Center for Animal Breeding (CENPALAB, Havana, Cuba). Animals were individually housed at the animals' facility of the Center for Genetic Engineering and Biotechnology, Havana, Cuba, and maintained under controlled environmental conditions and light cycles (12/12 hrs). Rats were fed with standard laboratory rodent's chow under no restriction. Following an acclimation week, the dorsum of the rats was conditioned to receive two controlled full-thickness wounds, under sodium pentobarbital (30 mg/kg) anesthesia. The cuts were generated with disposable 6 mm diameter punch biotomes (Acuderm, Ft. Lauderdale, USA). Two independent experiments were performed using the above described wound model. Thus, 10 rats (*n* = 20 wounds) were used for either GHRP-6 formulation or vehicle (1% CMC) groups in each experiment. Upon wounds induction the rats were randomly assigned to either group. The wounds were cleansed daily with saline, their contours traced on transparent plastic sheets and treated accordingly. Treatments were topically applied twice a day at the same hours during four days. Wounds closure dynamic was measured by planimetric analysis as described previously [16] using the ImageJ software, version 1.46r. Since the GHRP-6 intervention increased the rate of closure, the animals were terminated by anesthesia overdose on day five after wounding. Ulcers and a surrounding margin of intact skin (~5 mm) were collected and hemisectioned. One hemisection was preserved in RNA Later solution for further gene expression studies. The other hemisection was fixed in 10% buffered formalin, paraffin embedded, and 5-*μ*m sectioned. The specimens were stained with hematoxylin/eosin (H/E) and Mallory trichrome to examine collagen deposit. Other slides were destined for immunohistochemistry (as described below).

The impact of the treatment on the neodermal matrix reconstitution was qualitatively graded as described [17, 18]:

(0) Immature granulation tissue with a null or incipient formation of collagen fibrils, focally distributed with no alignment and not organized meshwork. Fibrin material prevails in the field. Mallory staining is detected in scarce foci.
(1) Scarce collagen fibrils suggestive of a primitive degree of organization, focally distributed, without horizontal alignment along the wound bed. Yet, fibrin occupies more than 50% of the field. Limited number of primitive neoformed vessels with empty lumen. Relative increase of positivity to Mallory staining.
(2) A general but coarse image of ECM granulation tissue accumulation, containing intermixed vertically and horizontally oriented collagen fibrils. Full replacement of fibrin by collagen. Fibrin has been fully replaced by collagen. Affinity to Mallory staining is observed.
(3) Complete ECM reconstitution, with mature and finely organized collagen fibrils horizontally deposited in the neodermis. The whole matrix appears positive to Mallory staining.

The number of infiltrating immunoinflammatory cells and neoformed vessels was determined within the granulation tissue of each wound. For this purpose, images of at least 10 microscopic fields (10–20x magnification) were captured and photographed so that mature vascular structures and infiltrated mononuclear cells were counted along with the assistance of the ImageJ processing system, version 1.46r.

### 2.4. CD31 Immunohistochemistry

Immunohistochemical determination of CD31 expression (platelet endothelial cell adhesion molecule-1, PECAM-1) was conducted as this is a marker protein of mature vascular endothelium [19]. Sections (5 *μ*m) were mounted on chromalum-coated slides, dewaxed, rehydrated, rinsed, and washed in PBS 1x solution for 30 min. Once endogenous peroxidase was quenched, the specimens were treated with target retrieval solution (Dako) equilibrated at 99°C. Tissue samples were then incubated for 40 min with 1/50 dilution of anti-CD31 antibody (Abcam 28364, USA) in background reducing solution (Dako). The immunohistochemical reactions were carried out using the labelled streptavidin/biotin-horseradish peroxidase conjugate method, according to the manufacturer's instructions (Dako). The peroxidase reaction was developed with diaminobenzidine and counterstained with hematoxylin.

### 2.5. Hypertrophic Scars Induction in Rabbit Ears

White male New Zealand rabbits (4.3–4.5 kg) were used in four independent and extemporaneous experiments. Three to four wounds were created on the ventral side of each ear, down to the surface of the cartilage, using a 6 mm diameter punch biotome (Acuderm) as described [20]. For the surgical procedures, rabbits were anesthetized with intramuscular ketamine (60 mg/kg) and xylazine (5 mg/kg). In order to ensure an exuberant scarring, the perichondrium was carefully scrapped with the surgical blade. The wounds were made on each side of the midline, avoiding the central ear artery and the marginal ear veins. In three experiments, rabbits were randomly assigned to either GHRP-6 (400 *μ*g/mL) treatment or 1% CMC placebo gel. The jelly solutions were administered using 1 mL sterile disposable syringes; 250 *μ*L was applied to each wound, which for the group of GHRP-6 represented an actual dose of 100 *μ*g per wound. Treatments were initiated immediately after surgery and continued thereafter until day 30, when most of the wounds had already completed reepithelialization.

The wounds were monitored and followed from day 14 until day 30 after wounding so as to detect the nodular firm consistency that precedes the clinical exuberance. The animals remained in observation for another 20 days after GHRP-6 administration had been completed. The incidence of firm, protruded nodules with nipple-like appearance arising in resurfaced wounds was registered weekly until day 50. After euthanasia (anesthesia overdose), the samples were collected in block, longitudinally bisected along the largest point of nodular growth. One hemisection was nitrogen frozen for additional studies and the other one was fixed in 10% neutral buffered formaldehyde and processed for histology. Five-micrometer sections were stained with H/E staining. Scar overgrowth was measured using the previously described scar elevation index (SEI) based on the cross-sectional scar area to the area of tissue excised to induce the wound [21]. Blinded researchers measured the sections using the ImageJ software package, version 1.46r.

### 2.6. Gene Expression Analysis

Total RNA was purified according to TRI Reagent standard procedure (Sigma, USA), following digestion with RQ1 DNase I (Promega, USA) to remove contaminating genomic DNA. Afterward, 500 ng of DNA-free RNA was reverse transcribed using Omniscript RT kit (Qiagen, Germany) with oligo-dT primer. The RT reaction was performed at 42°C for 60 min. PCR mixtures contained 1 *μ*L cDNA, 1 *μ*L of each primer (10 *μ*M), and 12.5 *μ*L 2x Taq MasterMix (Qiagen, Germany) in a final volume of 25 *μ*L. Specific sense and antisense primers, annealing temperatures, and number of repeating cycles for both studies are referred to in Table 1. Amplifying conditions were performed as follows: a first step of 95°C for 5 minutes, thereafter repeating cycles comprised of 95°C for 30 seconds, specific annealing temperature for 30 seconds and 72°C for 30 seconds, and a final extension step of 5 minutes at 72°C. PCR bands (8 *μ*L of PCR product plus 2 *μ*L of gel loading buffer) were resolved on 1.5% (w/v) agarose gel electrophoresis and visualized under ultraviolet light subsequent to being stained with ethidium bromide. PCR products were quantified using the Kodak ID 3.6 software package (Kodak Inc, USA). Beta-2 microglobulin was used as housekeeping gene for normalization.

### 2.7. Statistical Analysis

Statistical analyses were carried out using GraphPad Prism 6 for Windows, version 6.01. For clinical response, histomorphometric parameters, and gene expression data, normal distribution (Kolmogorov-Smirnov) and variance homogeneity (Brown-Forsythe) tests were performed. Once normality was demonstrated, differences between GHRP-6-treated and placebo-treated animals were determined using two-tailed unpaired Student's *t*-test. For non-Gaussian distributed data, Mann-Whitney* U* test was performed. For analyzing closure kinetics of rat wounds, two-way ANOVA was performed, followed by Sidak's multiple comparisons test. In all cases, values of *p* < 0.05 were considered statistically significant. The values shown represent mean ± SD (error bars).

## 3. Results

### 3.1. GHRP-6 Reduced Inflammation Markers and the Expression of Profibrogenic Cytokines in Normal Wounds

**Figure 1:**
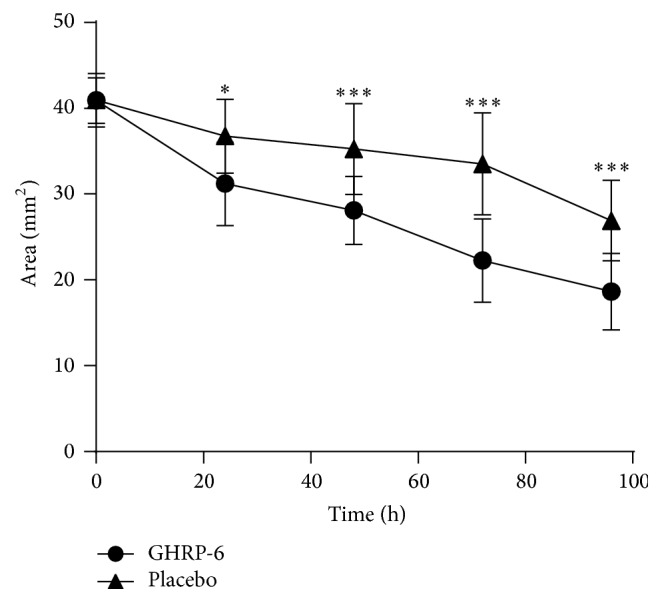
GHRP-6 accelerated wound closure. Differences in wounded area reduction appeared since the first 24 hours of postinjury. GHRP-6-induced contraction remained stable until hour 96, when the animals were terminated. Two-way ANOVA (^*∗*^ *p* = 0.016, ^*∗∗∗*^ *p* < 0.001).

As shown in Figure 1, GHRP-6 administration significantly enhanced wound closure as compared to 1% CMC placebo solution. Differences were noted after the first 24 hours (*p* = 0.016) following the initial administrations, which continued thereafter until the end of the experiment (*p* < 0.001).

**Figure 2:**
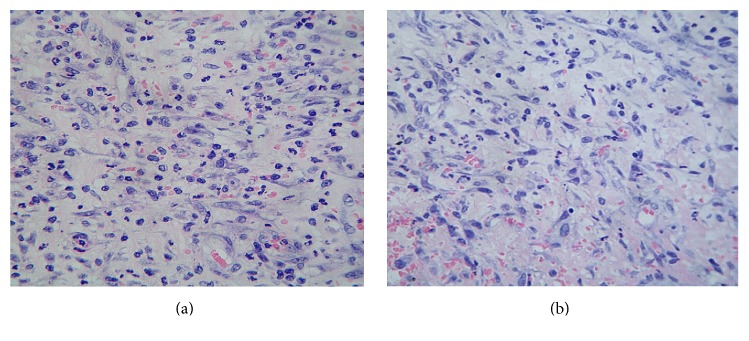
GHRP-6-mediated response to inflammation. Images are representative of (a) wounds topically treated with vehicle (1% CMC); (b) wounds topically treated with GHRP-6. GHRP-6 treatment reduced the inflammatory infiltration of mononuclear basophilic round cells. In contrast, CMC-treated wounds exhibit a physiologically normal infiltration, which matches the biological stage of the wound. 5 *μ*m section, H/E, 20x magnification.

At the histological analysis, and from a qualitative perspective, these wounds appeared less inflamed and with a higher degree of ECM organization, given by far less fibrin accumulation and thinner and horizontally distributed collagen bundles. Vessels were also aligned with the collagen fibers. Thus, the treatment not only reduced the wound area but also appeared to be associated with differences in the quality of the ECM as the inflammatory infiltrate.  Figure 2(a) is representative of the GHRP-6 effect on the inflammatory response, illustrating the reduction of infiltrated cells as compared to placebo-treated wounds (Figure 2(b)).

**Figure 3:**
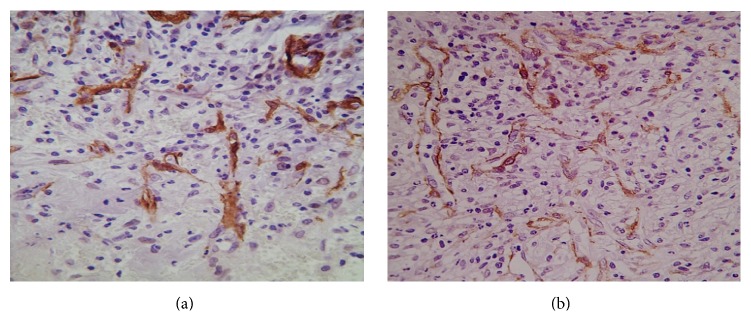
Impact of GHRP-6 treatment on wound angiogenesis. Anti-CD31 immunolabeling for mature endothelial cells. Images are representative of (a) vehicle (1% CMC)-treated wounds; (b) GHRP-6-treated wounds. No histological differences were detected between the groups in relation to the number of neovessels, their structure, distribution, organization, or CD31 positivity.

Since CD36 is implicated in angiogenesis regulation, special attention was addressed to the population of neovessels as to their general morphology. By routine staining, we ascertained that GHRP-6 treatment did not reduce the number of vessels, which also exhibited normal structure, organization, and distribution. Furthermore, CD31 expression was detected in all these vascular structures suggesting mature angiogenesis. Conclusively, GHRP-6 administration did not hinder wound angiogenesis in any respect (Figure 3(a)), as compared to placebo-treated wounds (Figure 3(b)). These histological findings support the scoring on the ECM maturation and the quantification of inflammatory cells across the wounds (Table 2).

**Figure 4:**
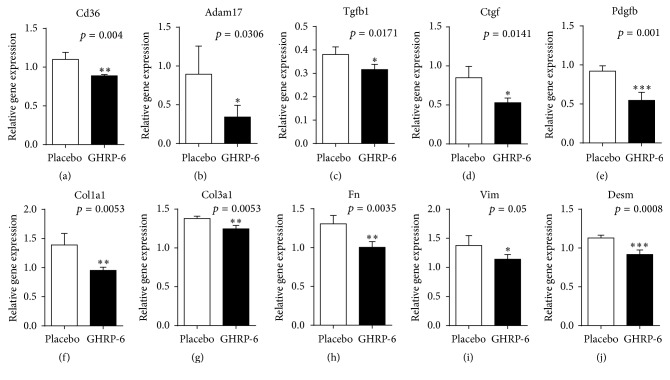
Influence of GHRP-6 on the expression of different gene families. RT-PCR experiments demonstrate the GHRP-6-induced reduction of the expression of its own receptor (Cd36). Concurrently, the peptide significantly reduced proinflammatory and profibrogenic cytokines. It is likely that the attenuation of these fibrogenic growth factors accounted for a reduction of extracellular matrix proteins and mesenchymal cells cytoskeleton proteins. Unpaired *t*-test (^*∗*^ *p* < 0.05, ^*∗∗*^ *p* < 0.01, and ^*∗∗∗*^ *p* < 0.001).

Following the preliminary histological data, suggesting a reduction of wound inflammation and a far more organized ECM, we addressed the gene expression study toward inflammatory and profibrogenic markers. We primarily examined Cd36 expression following topical GHRP-6 application and found that peptide reduced its receptor expression (*p* = 0.004) (Figure 4). Furthermore, the treatment significantly reduced Adam17 expression (*p* = 0.0306) and approached to significantly reduce Tnf (*p* = 0.07), which may partially contribute to explaining the substantial reduction of infiltrated inflammatory cells within the wound bed (Figure 4).

Furthermore, the most potent profibrogenic growth factors: Tgfb1, Pdgfb, and Ctgf also appeared significantly underexpressed in the GHRP-6-treated wounds (all *p* < 0.05) (Figure 4). In line with this, we observed a significant reduction in the expression levels of Col1a1 and Col3a1 (Figure 4, both *p* < 0.01). Concomitantly, we addressed the attention to filamentous and contractile proteins associated with fibroblasts and other differentiated mesenchyme-derived cells. Acta2 appeared close to a significant reduction (*p* = 0.06), whereas Des, Vim, and Fn transcriptional expression appeared significantly reduced (all *p* < 0.05), as compared to placebo-treated wounds.

### 3.2. GHRP-6 Prevented the Onset of HTS in Rabbits

According to pilot studies, our group determined that 400 *μ*g/mL represented an optimal dose level by reducing inflammation, promoting collagen fibers alignment, while aborting the onset of HTS in rabbit ears. A lower dose (200 *μ*g/mL) did not prevent the exuberant phenotype whereas a higher dose (800 *μ*g/mL) delayed reepithelialization in rats and rabbits (data not shown).

**Figure 5:**
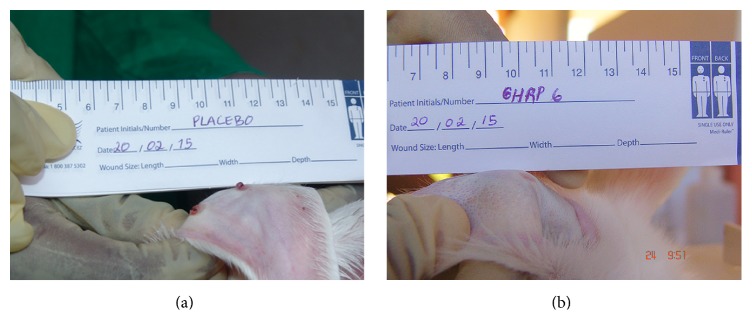
Topical GHRP-6 improved the macroscopic aspect of the wounds. (a) Representative wounds that evolved to hypertrophic scars (HTS). (b) Representative image of the effect of GHRP-6 administration.

Placebo-treated wounds appeared hypertrophied and proved a firm consistency by day 17 onward. For the three experiments, day 30 following injury established a clear definition on the wounds evolution. The most remarkable effect of GHRP-6 intervention can be ascribed to HTS prevention. As shown in Table 3, GHRP-6 administration aborted the debut of HTS in 90.5% of the treated wounds. These wounds were also negative to palpation. On the contrary, 87.5% of the wounds receiving the jelly CMC solution evolved to HTS with nipple-like, reddish appearance and a firm consistency nodule at palpation (Figures 5(a) and 5(b)).

**Figure 6:**
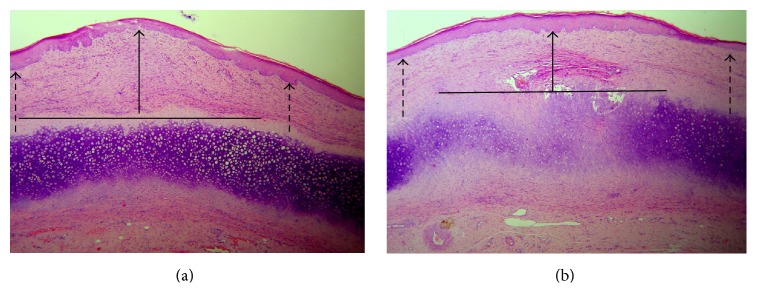
Microscopic aspect of the rabbits' ears wounds. (a) Representative image of “nipple” in which, above the cartilage and the perichondrium, there is a prominent accumulation of extracellular matrix. (b) Representative image of the effect induced by the GHRP-6 intervention. Note the reduction of extracellular matrix accumulation within the injured area. The “flattening” aspect is indicated by the solid line arrow. The dotted arrows indicate that the elevation within the center of the scar is similar to the adjacent intact skin. Images suggest that GHRP-6 reduced the local hypercellularity associated with the cartilage cells response. H/E 10x magnification.

The qualitative microscopic analysis of the GHRP-6 responsive wounds indicated that the peptide seems to primarily reduce both local hypercellularity associated with the cartilage perichondrium cells and the resulting ECM accumulation (Figures 6(a) and 6(b)). Accordingly, their SEI (1.12 ± 0.11) appeared largely different (*p* = 0.001) as compared to the placebo samples group (1.62 ± 0.15). It is notorious, however, that those GHRP-6 nonresponsive wounds (*n* = 8) that evolved to HTS exhibited similar microscopic appearance (not shown) and SEI values as compared to placebo control wounds (Table 3).

**Figure 7:**
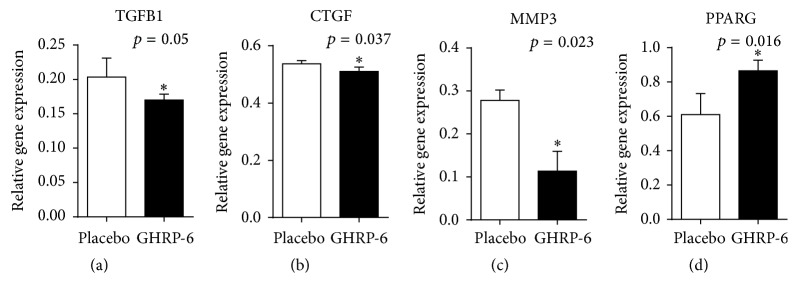
Potential bases of GHRP-6-mediated antifibrotic effect. Among these four genes significantly modulated by GHRP-6 of biological relevance within this realm are TGFB1 reduction and PPARG increase. Mann-Whitney* U* test. ^*∗*^ *p* < 0.05.

RT-PCR experiments shed light on the molecular mechanisms by which GHRP-6 appeared to modulate the fibrotic response. Among the genes studied (Table 1), GHRP-6 proved to significantly reduce TGFB1 and CTGF (*p* < 0.05) expression, with no effect on PDGFB gene expression. An unexpected finding was that MMP3 appeared significantly reduced in the GHRP-6-treated wounds (*p* = 0.02). Most meaningfully is that PPARG expression became significantly elevated with GHRP-6 treatment (*p* = 0.016), as compared to placebo-treated wounds (Figure 7).

## 4. Discussion

The evidence derived from these experiments supports the notion that CD36 is an active and approachable receptor to modulate the healing process. Here we have observed that CD36 occupation by GHRP-6 attenuates wound inflammation, accelerates wound closure, and above all improved wound's esthetic outcome by impacting ECM proteins accumulation. To our knowledge these findings are unprecedented for GHRP-6 within the context of cutaneous healing.

The experiment in rats, based on clean full-thickness controlled wounds, indicated that GHRP-6 pharmacodynamics has likely involved attenuation of immunoinflammatory mediators, their effector cells, and the reduction of fibrosis-inducing cytokines. The concerted action of these two elemental mechanisms may have theoretically translated into a particular modulation of fibroblasts response to injury, leading to precocious closure with a reduced scarring. Outstandingly, the mechanisms underlying this pattern of healing do not appear to interfere with the angiogenic repopulation nor with the reepithelialization process.

The response of these wounds reminds us of the pattern of healing described for MG53 protein (a membrane repair machinery member), so that the treatment facilitated wound healing along with a reduced scarring in rodent models. This antiscar effect was explained by interfering with TGF-*β*-dependent activation of myofibroblasts differentiation and reduction of ECM proteins accumulation [22]. Similarly, antiscarring healing properties are described for plants' principles that downregulate the expression of fibrogenic-related molecules such as TGF-*β*1 and the downstream events, leading to fibrosis and scar formation [23]. In addition to a direct action of GHRP-6 on TGFB1 gene expression, we deem that the reduction of inflammatory effectors could have also contributed to enhancing the healing process and to reducing fibrosis. In an animal model of liver ischemia/reperfusion, we previously demonstrated that GHRP-6 prevented internal organs parenchymal activation and the onset of a systemic inflammatory response syndrome by downregulating proinflammatory cytokines [24]. Subsequent studies have demonstrated the ability of different GHRPs to ameliorate local and systemic inflammatory processes in a variety of experimental scenarios by suppressing the activation of NF-*κ*B, the consequent expression of proinflammatory cytokines, and acting as chemokine receptor antagonist [25–27]. Differentiation to myofibroblasts, collagen fibrillogenesis, and matrix accumulation are controlled by opposing forces: proinflammatory and profibrogenic, that require a fine tuning to ensure a proper esthetic healing and effective mechanical properties of the ECM [28, 29]. The overall interpretation of the data from (i) the rate of closure, (ii) microscopic appearance of the collagen fibrils alignment/organization, (iii) impact of the treatment on the transcriptional expression of cytoskeleton filamentous proteins (smooth muscle *α*-actin (*α*-SMA), desmin, and vimentin) supports the hypothesis that, in this context, GHRP-6 has shifted the balance toward “a more regenerative” rather than a reparative phenotype.

Aside from the limitations of this work to fully elucidate the underlying mechanism by which GHRP-6 mediated the refinement of the wounds fibrogenesis in the rats experiment, an important contribution is the unprecedented evidence that the peptide reduced the onset of HTS in the rabbit's ear model. This represents an extension of the GHRP-6 antifibrotic potential demonstrated years ago by our group in an animal model of liver fibrosis [7]. Nevertheless, and in contrast to the liver fibrosis data, we have no evidence that GHRP-6 is able to revert the consolidated HTS following repeated experimental attempts. Thus, the reproducible findings regarding GHRP-6-mediated HTS prevention are based on the immediate and consecutive administration of the molecule once the injury is induced.

The mechanisms supporting the GHRP-6-mediated HTS prevention may be related to a potential modulation of the fibrogenic response, especially by TGF-*β*1 transcriptional deactivation and its downstream effector CTGF, as has been previously described [30]. Nevertheless, we have not elucidated the pathways involved in the GHRP-6-mediated TGFB1 gene expression reduction. Under these circumstances, we have reproducibly observed [7] that GHRP-6 increases PPARG expression which may have counteracted TGF-*β*1-associated fibrogenic input. The fact that CD36 occupation by GHRP-6 upregulates PPARG gene expression is noteworthy in this context and represents an additional pharmacologic property for this peptide. Although the molecular pathways underlying the antifibrotic effects of PPAR*γ* remain elusive, an antagonistic relationship is proposed between PPAR*γ* and TGF-*β*1 signaling in fibrosis. For more than a decade ago, PPAR*γ* has been reputed as a fibrosis-response regulating factor and its activation represents an innovative pathway to control fibrotic diseases [31, 32].

Taking into account the broad spectrum of TGF-*β*1 physiology in the fibroblasts/myofibroblasts differentiation events [33], we deem that the reduction of the local scar cellularity and perichondrial matrix accumulation in those animals receiving GHRP-6 could be attributable to TGFB1 transcriptional and functional switch-off. Since the predominant microscopic aspect of the GHRP-6-treated wounds was characterized by meagre cartilage scars, slimmer perichondrium membranes, and far less active cells, we hypothesize that the peptide somehow attenuates the perichondrial activation response to the trauma and/or a possible mesenchyme-to-mesenchyme redifferentiation process, thus lessening the surge of fibroblast and myofibroblasts. In line with this notion, we had documented that GHRP6 prevented hepatic stellate cells activation by reducing CD68, *α*-SMA, and vimentin local expressions. All these events could be primarily presided by the GHRP-6-related reduction of TGFB1 and CTGF expression in both parenchymal and nonparenchymal cells [7].

## 5. Conclusions

The evidence described here presupposes the existence of a GHRP-6/CD36-mediated anti-inflammatory and antifibrotic loop that appears to improve wound closure and esthetic. The activity of this binomium may represent a novel and attractive avenue toward the timely prevention of dismal cutaneous processes such as keloids and HTS.

## Figures and Tables

**Table 1:** Genes in study and PCR amplification data.

Symbol	Name	Gene Bank accession number		Sequence	Annealing temp. (°C)	Number of cycles	Product length (bp)
*Rat oligonucleotides*							
Col1a1	Collagen, type I, alpha 1	NM_053304	Sense antisense	CCCTCTGTGCCTCAGAAGAACT GCCAGTCTGTTGGTCCATGTAG	58	30	234
Col3a1	Collagen, type III, alpha 1	NM_032085	Sense antisense	AAGAGCGGAGAATACTGGGTTG CAGGATTGCCATAGCTGAACTG	58	30	214
Tgfb1	Transforming growth factor, beta 1	NM_021578	Sense antisense	TGCCAGAACCCCCATTGCTG TCCACCTTGGGCTTGCGACC	70	35	700
Ctgf	Connective tissue growth factor	NM_022266	Sense antisense	AGAGCTGGGTGTGTGTCCTCC GCAGCAAACACTTCCTCGTGG	70	30	547
Acta2	Actin, alpha 2, smooth muscle, aorta	NM_031004	Sense antisense	GTGCCTATCTATGAGGGCTATGCTCTGC CATACTCCTGTTTGCTGATCCACATCTGC	68	35	601
Tnf	Tumor necrosis factor	NM_012675	Sense antisense	ATGGCATGGATCTCAAAGACAA TCTCCTGGTATGAAGTGGCAAA	58	40	150
Adam17	ADAM metallopeptidase domain 17	AJ012603	Sense antisense	GTGACATGAATGGCAAATGTGA TGGACAAGAATGCTGAAAGGAA	55	40	172
Cd36	CD36 molecule (thrombospondin receptor)	NM_031561	Sense antisense	GTCGTATGGTGTGCTGGACATT TGGCTTGACCAGTATGTTGACC	62	35	217
Vim	Vimentin	NM_031140	Sense antisense	GGATTTCTCTGCCTCTTCCAAA GTCATCGTGGTGCTGAGAAGTC	62	35	164
Des	Desmin	NM_022531	Sense antisense	TCCGTGCTCAGTATGAGACCAT GCATCAATCTCGCAGGTGTAGG	62	35	181
Pdgfb	Platelet-derived growth factor beta polypeptide	NM_031524	Sense antisense	AACATGACCCGAGCACATTCT TGGCTTCTTTCTCACAATTTCG	62	35	303
Fn1	Fibronectin 1	NM_019143	Sense antisense	GTGGTCATTTCAGATGCGATTC GGCTCCGAGATACTCTTTCTGC	62	35	227
B2m	Beta-2 microglobulin	NM_12512	Sense antisense	CGGTGACCGTGATCTTTCTGGT GGTGACGGTTTTGGGCTCCTT	58	30	332

*Rabbit oligonucleotides*							
TGFB1	Transforming growth factor, beta 1	XM_008249704	Sense antisense	TCATTTACCGTCACCTGGATTG TGTGTAGATGTTGAGCCCGTTC	72	40	229
PDGFB	Platelet-derived growth factor beta polypeptide	XM_008257019	Sense antisense	GGTGAGAAAGATCGAGATTGTGC GTGTGCTTGAACTTGTGGTGCT	62	40	231
CTGF	Connective tissue growth factor	XM_008263527	Sense antisense	GGGCTAAGTTCTGCGGAGTATG CATTGTCCCCAGGACAGTTGTA	62	30	162
MMP3	Matrix metallopeptidase 3 (stromelysin 1, progelatinase)	NM_001082280	Sense antisense	TCTCTTCCTTCAGCAGTGGATG TTCCTTATCAGAAATGGCAGCA	58	40	186
COL1A1	Collagen, type I, alpha 1	XM_008271783	Sense antisense	CCTGGGCAGAGAGGAGAAAGAG CCTCACGTCCAGATTCACCAG	62	30	157
IGFBP3	Insulin-like growth factor binding protein 3	NM_000598 (human)	Sense antisense	TGCCGTAGAGAAATGGAAGACA GCCCATACTTATCCACACACCA	72	40	172
PPARG	Peroxisome proliferator-activated receptor gamma 1	AY166780	Sense antisense	TGATGAATAAAGACGGGGTCCT CCACTGAGAATGATGACGGCTA	62	40	187
P4HB	Prolyl 4-hydroxylase, beta polypeptide	NM_001171047	Sense antisense	ATGACCAAGTACAAGCCCGAGT GCGTAGAACTCCACGAAGACG	62	30	212
COL3A1	Collagen, type III, alpha 1	XM_002712333	Sense antisense	AAAGAAAGCCCTGAAGCTGATG CCACCAATATCATAGGGTGCAA	62	30	195
B2M	Beta-2 microglobulin	XM_002717921	Sense antisense	CGCCCCAGATTGATATTGAGTT GATCCCATTTCACTGTCATAGGC	62	30	195

**Table 2:** Impact of GHRP-6 topical administration on inflammation and fibroangiogenesis.

	Inflammatory cells	Active vessels	Dermal matrix reconstitution
GHRP-6	7.86 ± 2.41^*∗*^	8.34 ± 3.02	1.9 ± 0.36^*∗*^
Placebo	15.74 ± 3.91	8.38 ± 2.89	2.49 ± 0.38

^*∗*^ *p* = 0.001. Two-tailed unpaired Student's *t*-test.

**Table 3:** Effect of GHRP-6 in HTS prevention.

Group	Total # wounds	Hypertrophic phenotype	Normal phenotype	SEI	SEI^&^
GHRP-6	84	8 (9.5%)	76 (90.5%)	1.12 ± 0.11^*∗*^	1.63 ± 0.44
Placebo	80	70 (87.5%)	10 (12.5%)	1.67 ± 0.15	1.66 ± 0.36

^&^SEI: scar elevation index measured in 8 nonresponsive wounds of the GHRP-6 treated group. ^*∗*^ *p* = 0.001; two-tailed unpaired Student's *t*-test.
